# Social Capital, Income Loss, and Psychobehavioral Responses amid COVID-19: A Population-Based Analysis

**DOI:** 10.3390/ijerph17238888

**Published:** 2020-11-29

**Authors:** Tsz Wai Li, Tatia Mei-chun Lee, Robin Goodwin, Menachem Ben-Ezra, Li Liang, Huinan Liu, Wai Kai Hou

**Affiliations:** 1 Centre for Psychosocial Health, The Education University of Hong Kong, Hong Kong, China; twli@eduhk.hk (T.W.L.); lleunglik@gmail.com (L.L.); s1107186@s.eduhk.hk (H.L.); 2 State Key Laboratory of Brain and Cognitive Sciences, The University of Hong Kong, Hong Kong, China; 3 Laboratory of Neuropsychology and Human Neuroscience, The University of Hong Kong, Hong Kong, China; 4 Department of Psychology, University of Warwick, Coventry CV4 7AL, UK; Robin.Goodwin@warwick.ac.uk; 5 School of Social Work, Ariel University, Ariel 40700, Israel; menbe@ariel.ac.il; 6 Department of Psychology, The Education University of Hong Kong, Hong Kong, China

**Keywords:** depression, social capital, income loss, preventive behaviors, COVID-19

## Abstract

This study examined the associations of perceived social capital and income change since the outbreak with probable depression and preventive behaviors during the COVID-19 pandemic in Hong Kong. Random digit dialing recruited a population-representative sample of 3011 Hong Kong Chinese aged ≥ 15 years (mean = 44, 55% females) between February 25 and April 29 2020. Respondents reported social capital (perceived interpersonal trust, social harmony, and sense of belonging), income change since the outbreak (loss vs. gain/no change), depressive symptoms, preventive behaviors, and demographics. Controlling for sociodemographics, lack of perceived interpersonal trust was associated with probable depression and avoiding contact with people with respiratory symptoms. Lack of perceived sense of belonging was associated with probable depression and decreased odds of adopting preventive behaviors. Lack of perceived social harmony was associated with probable depression and increased odds of used face masks among respondents with income loss only. Our results suggest that social capital is related to lower risk of depression and to higher chance of used face masks particularly among those experiencing income loss related to COVID-19. Prevention of mental health problems and promotion of effective preventive behaviors could be implemented by focusing on support for those who are socioeconomically disadvantaged.

## 1. Introduction

The Coronavirus Disease 2019 (COVID-19) pandemic is regarded as the biggest global health crisis in recent decades. Overseas cases first appeared in Asia and spread to Europe and the U.S.A. within months [1], with more than 188 countries affected and 10 million people infected with COVID-19. Stressors related to COVID-19 could contribute to widespread emotional distress and increased risk of psychiatric conditions across populations [2]. In a nationwide sample of Chinese (*n* = 52,730), 35% had experienced psychological distress during the COVID-19 pandemic [3]; a further sample (*n* = 3480) collected at the initial stage of COVID-19 in Spain showed 21.6% reported probable anxiety, 18.7% probable depression, and 15.8% moderate to high levels of post-traumatic stress disorder (PTSD) symptoms [4]. Prevalence of probable anxiety and depression was associated with absence of new preventive routines such as using a face mask when they go out and disruption to regular daily routines such as healthy eating, sleep, and leisure activities in large population-representative samples of a region [5].

The high transmissibility of COVID-19 places a huge demand upon the medical systems of affected regions [6], with insufficient medical protection supplies and overburdened medical infrastructure and healthcare workers [2]. Countries have adopted different infection control measures for the COVID-19 pandemic based on emergency levels, including the introduction of lockdowns, closure of public facilities, and restrictions on business activities [7]. The current pandemic alters the daily routines of populations across the globe, such as reduced interpersonal interactions, working from home arrangements, and an additional adoption of preventive behaviors [8]. Such large-scale infection control works best within a well-functioning community in which a high degree of social capital facilitates coordination through social integration and consensus [9]. Manifestations of social capital are evident in community efforts at the early stage of mass pandemic, and supplement government aid [10,11].

Social capital should be understood with reference to the dual influence of Western and Chinese cultures on Hong Kong society due to its special historical background [12]. Early studies examining social capital suggested this was limited in Hong Kong, with low levels of participation in government bodies or other non-government institutions. However, subsequent analysis found high levels of social cohesiveness in Hong Kong, as reflected by public engagement in advocacy groups, informal support groups, and close communications with significant others [13]. Community efforts and mobilizations were especially salient in Hong Kong society during major disasters such as SARS in 2003 and anti-extradition bill protests in 2019, suggesting the importance of social factors in shaping people’s psychobehavioral responses [13,14]. Yet, there is a deficit of knowledge about the associations between social capital and psychobehavioral outcomes amid crises in Hong Kong.

Social capital can be divided into two types: cognitive and structural social capital. Cognitive social capital represents the personal, attitudinal components of social interactions, such as values, norms, beliefs, and attitudes, known as interpersonal trust and social cohesion. Structural social capital reflects the societal components of social interactions, such as group/community membership, participation in civic activities, or social networks [15]. Cognitive social capital is particularly important for mental health research during the current pandemic because previous evidence has shown that, compared to structural capital, cognitive social capital has stronger associations with mental health outcomes [15,16]. Therefore, this study focuses on cognitive social capital: trust, social harmony, and sense of belonging, which will hereafter be referred to as social capital in the current study.

### 1.1. Social Capital, Mental Health, and Preventive Behaviors

Subjective evaluations of social relationship (i.e., social connectedness) rather than objective interactions were found to be more closely related to mental health [17]. Individuals with higher social capital tend to perceive higher levels of social support from their social networks [18], and lower sense of helplessness and social isolation, which is related to reduced depression risk [16]. Social capital functions as a protective factor against psychological distress across disaster contexts, including displaced residents affected by the Fukushima Daiichi nuclear disaster [19], and women affected by the Deepwater Horizon oil spill [20]. Social capital is also crucial for facilitating coordination for mutual benefit throughout the process of handling disasters, from preparation at the initial stage to response and recovery [9]. Social capital during a pandemic can promote the exchange of valued resources such as information and support, and benchmark individual behaviors that facilitate collective actions, including preventive behaviors [10,21]. Collective efforts to combat the COVID-19 pandemic are important for reducing infection and transmission through promoting health behaviors [22], preventive care [21], and preventive behaviors [23,24]. Previous studies have consistently found a positive association between social capital and vaccination [25], suggesting that social capital could enhance and strengthen non-pharmacological preventive behaviors in the midst of an epidemic [23].

### 1.2. Resource Loss as the Moderator

Prior studies focused on differences in psychobehavioral outcomes of the population as functions of static socioeconomic status (SES) during both ordinary times and crises. Persons with low static SES may access proportionately less social capital relative to those with high static SES, as social capital tends to distribute coping resources in direct proportion to social status through social networks [26]. In the beginning of the lockdown for COVID-19 in the United Kingdom, people with low static SES were found to experience more psychosocial distress and difficulties in behavioral adjustment to the pandemic relative to those with higher static SES [27]. Among indicators of SES, income could be regarded as a dynamic coping resource for psychological and behavioral adaptation. COVID-19 has placed huge financial insecurity on the global population, with worldwide mass lay-offs and reductions in income potential [28]. Conservation of resources (COR) theory suggests that loss of personal, social, and material resources is a key factor in poorer psychological adaptation during trauma and chronic stress conditions, independent of one’s static SES [29,30,31]. Loss of financial resources during and after disasters can have a significant adverse impact on the affected populations’ coping and mental health, and at the same time increase their vulnerability to further and future resource loss [30]. Financial loss related to the aftermath of Hurricane Katrina was associated with poor mental health [32]. Disaster-induced change in SES (i.e., employment status or income change) has been associated with poor subjective health, controlling for education level as one of the common static SES indicators [33].

### 1.3. The Present Study

This study examined the associations of perceived social capital and income change since the outbreak with probable depression and preventive behaviors during the COVID-19 pandemic in Hong Kong. We expected that lack of perceived social capital would be associated with higher odds of probable depression and lower odds of preventive behaviors. Considering the ongoing changes in economic downturn during the current pandemic, we also stratified our analysis to examine the role of income change in the associations between perceived social capital and psychobehavioral outcomes. We expected that the inverse association between perceived social capital and probable depression and the positive associations between perceived social capital and preventive behaviors would be stronger among persons who experienced loss in income during the pandemic relative to those who did not.

## 2. Materials and Methods

### 2.1. Respondents and Procedure

Following approval from the Ethics Committee of the Education University of Hong Kong (2018-2019-0292), respondents were recruited by telephone surveys conducted by the Centre for Communication and Public Opinion Survey of the Chinese University of Hong Kong and Hong Kong Public Opinion Research Institute during the period February 25–April 29 2020. A total of 85 cases of COVID-19 were reported up to February 25. Limited public health intervention (e.g., school closures, limited government services) was implemented due to the low infection rate. However, beginning in March there was a rapid increase of COVID-19, cases reaching 1037 by end of April. Since then, more intensive infection control measures were imposed, such as tightened quarantine controls, enhanced testing, an entry ban on non-HK residents, and a prohibition on group gathering [34,35]. A computer-assisted telephone interview (CATI) system was employed. Random digit dialing was conducted based on a dual-frame sampling approach with both landline and mobile phone numbers (50% each) drawn from the databases released by the Hong Kong Communication Authority. All eligible respondents were Hong Kong Chinese, 15 years of age or older, and Cantonese-speaking. For the landline phone calls, when there were multiple eligible members in a successfully contacted household, the one with the closest birthday to the interview date was selected. Further attempts were made by CATI to dial numbers for those with responses as “no answer”, “busy”, or “eligible respondent not at home”. Oral informed consent was obtained before each interview started. All interviews were conducted from 2 pm to 10 pm on both weekdays and weekends. A total of 84,709 telephone numbers were attempted, 38,468 (45.4%) of them were ineligible for interview (i.e., invalid, non-resident/business telephone, fax numbers, no eligible respondent) and 42,025 (49.6%) were unconfirmed eligible. Among the 4216 (5.0%) eligible, interviews were completed for 3011 (71.4%); 882 (20.9%) refused, and 323 (7.7%) eligible respondents did not complete the interviews. A response rate of 71.4%, defined by (Completed divided by Eligibles), was recorded (sampling error = ±2.2% (95% confidence level)). The participation and nonparticipation rates were acceptable and comparable with the population-representative samples in prior studies in Hong Kong [31,36,37].

### 2.2. Measures

#### 2.2.1. Demographics

We asked respondents’ age in years, gender, marital status, education level, employment status, and monthly household income. Respondents also reported any income change since the COVID-19 outbreak: gain/no change (0) vs. loss (1).

#### 2.2.2. Social Capital

Three cognitive social capital items were drawn from the short version of Adapted Social Capital Assessment Tool (SASCAT) [38,39] assessing perceived social capital: perceived interpersonal trust, social harmony, and sense of belonging on a 4-point scale (1 = strongly disagree, 2 = disagree, 3 = agree, and 4 = strongly agree). Each item was recoded into “presence” (strongly agree/agree = 0) or “lack of” (strongly disagree/disagree = 1). 

#### 2.2.3. Probable Depression

Depressive symptoms in the previous two weeks were assessed using the Chinese 9-item Patient Health Questionnaire (PHQ-9) [40] on a 4-point scale (0 = not at all, 1 = on several days, 2 = on more than half of the days, 3 = nearly every day). Alpha in the current study was 0.86. The total scores were recoded into 0 (scores = 0–9) or 1 (scores = 10–27) for probable depression [31,41].

#### 2.2.4. Preventive Behaviors

Respondents reported whether or not (No = 0/Yes = 1), in the last two weeks, they had performed each of the following behaviors to prevent COVID-19 infection [42]: (1) used face masks, (2) washed hands more often, (3) avoided contact with people with respiratory symptoms, and (4) avoided going to crowded places. 

### 2.3. Statistical Analysis

Missing data (< 1%) was replaced by multiple imputations. The prevalence of probable depression, each preventive behavior, and different dimensions of perceived social capital were estimated with 95% confidence intervals (95% CIs). Sociodemographics were recoded and included in subsequent statistical analyses: age: 15–24 (0) vs. 25–34 (1), 35–44 (2), 45–54 (3), 55–64 (4), and 65 or above (5); gender: male (0) vs. female (1); marital status: married (0) vs. unmarried (1); education level: tertiary (0) vs. secondary (1) and primary or below (2); employment status: employed (0) vs. dependent (1) and unemployed (2); monthly household income: ≤HK$19,999 (4), HK$20,000–HK$39,999 (3), HK$40,000–HK$59,999 (2), and HK$60,000–HK$79,999 (1) vs. ≥HK$80,000 (0) (US$1≈HK$7.80). Income change was recoded into gain/no change (0) vs. loss (1).

Logistic regression models were used to examine the associations between perceived lack of social capital (“lack of” = 1) and the outcomes probable depression (PHQ-9 scores 10–27 = 1) and the adoption of each preventive behavior (Yes = 1), respectively, controlling for sociodemographics. Interaction terms tested the moderating effects of income change in the logistic regression models. The associations between perceived social capital and outcomes were stratified into income gain/no change (0) and income loss (1), respectively. Adjusted odds ratio (aOR) with 95% CI indicated the independent association of each correlate with an outcome. All analyses were performed using R software.

## 3. Results

### 3.1. Sample and Prevalence

The sample resembled the population in terms of age group distribution, gender, education level, and other demographics (Table 1) [43]. The prevalence of probable depression (PHQ-9) was 21.3% (95% CI = 19.86–22.79%). The most common preventive behaviors were: used face masks (> 97%) (95% CI = 97–98%) and washed hands more often (> 92%) (95% CI = 91–93%), followed by 79.7% (95% CI = 78–81%) avoided going to crowded places and 72.1% (95% CI = 70–74%) avoided contact with people with respiratory symptoms. The prevalence of perceived social capital was as follows: perceived interpersonal trust = 66.8% (95% CI = 65.14–68.50%), perceived social harmony = 54.9% (95% CI = 53.09–56.64%), and perceived sense of belonging = 96.6% (95% CI = 95.93–97.23%). Descriptive statistics are summarized in Table 2.

### 3.2. Multivariable Logistic Regressions

#### 3.2.1. Social Capital and Probable Depression

**Figure 1:**
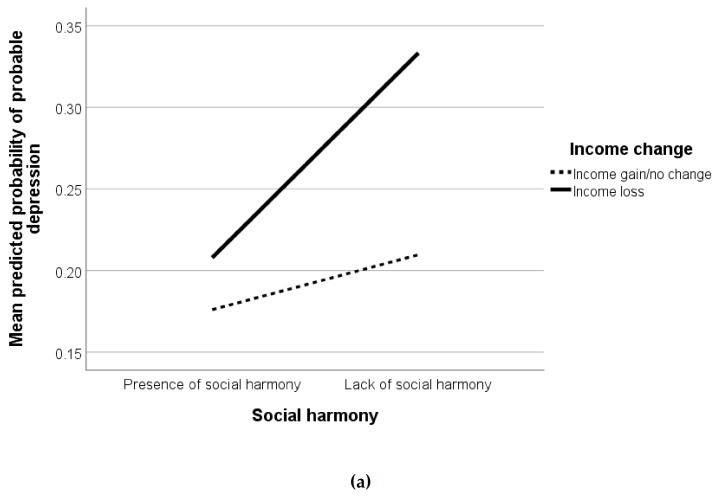

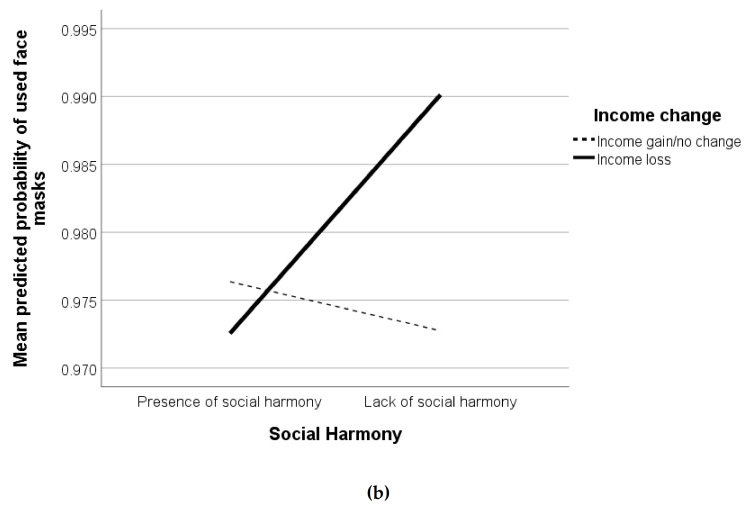
(**a**) Simple slope of the association between lack of social harmony and probable depression moderated by income change; (**b**) Simple slope of the association between lack of social harmony and used face masks moderated by income change.

Controlling for sociodemographic covariates (Table 3), lack of perceived interpersonal trust and lack of perceived sense of belonging were positively correlated with probable depression (aOR = 1.58, 95% CI = 1.29–1.93; and aOR = 2.53, 95% CI = 1.66–3.84, respectively). The association between perceived social harmony and probable depression differed between the two income change groups (loss vs. gain/no change) (*p* = 0.026) (Table A1). Lack of perceived social harmony was positively related to probable depression among respondents who experienced income loss (aOR = 1.58, 95% CI = 1.14–2.21), whereas the positive association was not significant among those with gain/no change in income (aOR = 0.99, 95% CI = 0.77–1.27). A simple slope test is shown in Figure 1a.

#### 3.2.2. Social Capital and Preventive Behaviors

Controlling for sociodemographic covariates, lack of perceived interpersonal trust was positively associated with avoided contact with people with respiratory symptoms (aOR = 1.28, 95% CI = 1.06–1.56), whereas lack of perceived sense of belonging was associated with not used face masks, not washed hands more often, not avoided contact with people with respiratory symptoms, and not avoided going to crowded places (aOR = 0.31–0.58, 95% CI = 0.13–0.37, 0.73–0.91) (Table 3). The positive association between lack of perceived social harmony and used face masks differed between respondents with income loss and those with gain/no change in income (*p* = 0.043) (Table A1). The association was significant only among respondents who reported income loss (aOR = 4.08, 95% CI = 1.21–13.75) but not those reporting gain/no change in income (aOR = 0.99, 95% CI = 0.53–1.87). A simple slope test is shown in Figure 1b.

**Figure 2:**
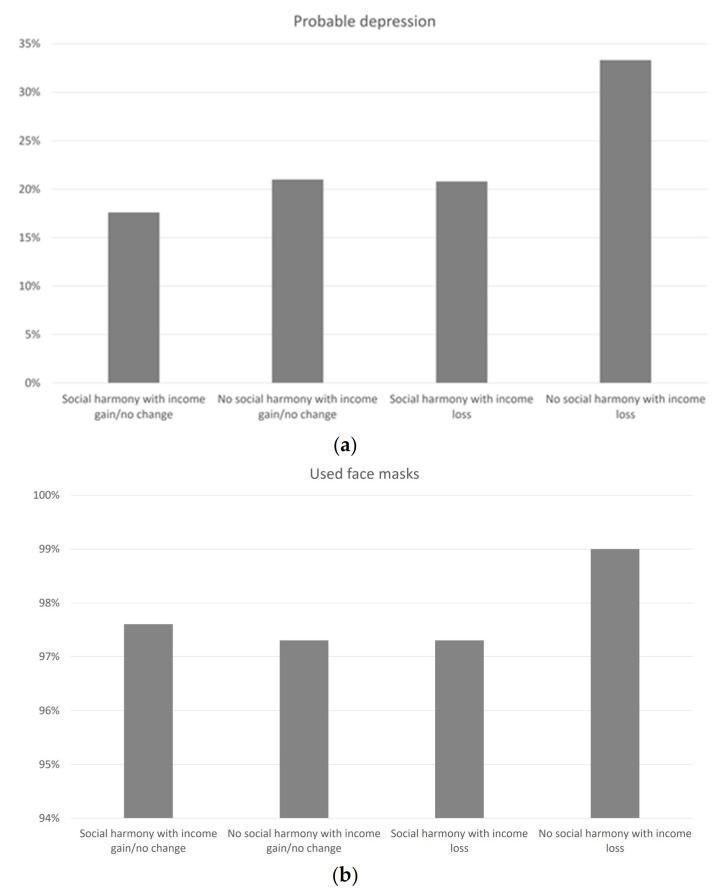
(**a**) Prevalence (%) of probable depression across different groups of social harmony and income change; (**b**) Prevalence (%) of used face masks across different groups of social harmony and income change.

Figure 2a,b shows the stratification of probable depression and preventive behaviors across different groups of social harmony and income change. The highest percentages of probable depression and used face masks were observed among respondents perceiving no social harmony who experienced income loss since the outbreak. The percentages were similar between respondents perceiving no social harmony but with gain/no change in income and those perceiving social harmony and with income loss. All descriptive and inferential statistical analyses were replicated with the sample aged 18 years or above (*n* = 2921) and showed highly consistent findings (Supplementary Tables S1 and S2).

## 4. Discussion

The aim of this study was to investigate the associations of perceived social capital with probable depression and preventive behaviors during the early months of the COVID-19 pandemic in Hong Kong. Analyzes were stratified by income change (loss vs. gain/no change) with consideration of the financial recession that may follow the outbreak of COVID-19. Lack of perceived interpersonal trust and perceived sense of belonging were associated with higher odds of depression. The positive association between lack of perceived social harmony and probable depression was significant only among those who experienced income loss, and not those who reported gain/no change in income since the COVID-19 outbreak. Different dimensions of perceived social capital were related to both increased and decreased preventive behaviors. Lack of perceived sense of belonging was associated with not used face masks, not washed hands more often, not avoided contact with people with respiratory symptoms, and not avoided going to crowded places. Lack of perceived interpersonal trust was positively associated with avoided contact with people with respiratory symptoms. The positive association between lack of perceived social harmony and used face masks was significant among people with income loss but not those with gain/no change in income.

Disasters can increase the vulnerability of a population, while social capital fosters recovery and adaptation [10]. Our findings are in line with previous evidence on the buffering role of social capital against psychological distress across disaster settings [19,20]. Among victims displaced from Fukushima following the nuclear disaster after the Great East Japan Earthquake, social capital (perceived interpersonal trust and social participation) was associated with reduced anxiety and general distress [19]. Perceived sense of community was related to lower depressive symptoms among people affected by the Deepwater Horizon oil spill [20]. There is evidence showing that social capital facilitates positive emotional and instrumental support and thus more positive cognitive appraisal of stressors that jointly reduce psychological stress [44].

Communities with high social connections permit the exchange and transfer of knowledge, adaptive resources, and the capacity for coping behaviors—all of which are crucial for encouraging preventive behaviors during hazards and crises [45]. Social capital can also be seen as social pressure that benchmarks preventive behaviors [21]. Influenza vaccination was more common among persons perceiving higher social cohesion [21,24]. Consistent with previous evidence [23], the current study found that a perceived sense of belonging was associated with higher chance of taking preventive behaviors.

Perceived interpersonal trust nonetheless was associated with a lower chance of avoided contact with people with respiratory symptoms, contrary to prior evidence on the positive links between parents’ neighborhood trust and children’s vaccination [24] and between generalized state-level trust and immunization [25] during the H1N1 pandemic. One possible explanation for this is that high interpersonal trust refers to higher faith we have in people around us, and we are more likely to perceive lower risk of being infected with COVID-19 from fellow people and thus adopt less preventive behaviors [46]. Interpersonal trust may also explain the reliance on community support during the pandemic, which in turn could result in fewer preventive behaviors [46]. In line with this, a lack of perceived interpersonal trust could be related to higher perceived risk, which then encourages more preventive behaviors [46,47].

Persons with low static SES are susceptible to infection through low-skilled and manual jobs that cannot be replaced by working from home, and they are also likely to have lower access to quality health care and greater financial strain due to unstable work conditions and loss of income during COVID-19 [48,49,50]. In addition, during widespread social unrests in Hong Kong, people with lower static SES have been shown to be more vulnerable to political and economic uncertainty and reported higher probable depression relative to those with higher static SES [51]. We found that the associations between social capital and probable depression/preventive behaviors were moderated by change in SES, particularly income loss, controlling for static SES variables including education level and monthly household income. COR theory suggests that resource loss begets future loss [30], and thus we expected that persons experiencing income loss were also more likely to have less social capital for coping and thus poorer mental health, and were less able to use adaptive behavioral responses to the current pandemic. Our findings suggest a need to mitigate the potential mental health problems among people perceiving poor social harmony and experiencing income loss since the COVID-19 outbreak. The economic crisis that follows COVID-19 is expected to have a far-reaching impact on population health [52]. There is evidence showing that COVID-19 impacts different earning groups in the labor market differently, with persons of lower static SES more prone to income loss under pressure from the threat of mass layoffs, such as in the service industry [53]. Decline in financial resources during the period of financial uncertainty could then eventually lead to more devastating psychobehavioral effects among those with low static SES, who already suffered the most during the crises.

The use of face masks has been a widely accepted and practiced preventive behavior in Hong Kong since the outbreak of SARS in 2003, and was enforced by law during this pandemic [11]. Although we did not assess attitude towards preventive behaviors, it was assumed that individuals adopt preventive behaviors based on their positive appraisals towards the effectiveness of those behaviors [54]. However, we found that while lack of perceived social harmony was related to higher chance of using face masks, this was evident among respondents with income loss but not those with gain/no change in income. One possible explanation is that persons who have been experiencing income loss are likely to be more motivated to adopt preventive behaviors in order to avoid further potential resource loss [30]. This indicates the importance of assessing behavioral outcomes in addition to mental health in the current pandemic.

### Limitations

Some limitations should be noted in interpreting the current findings. First, the cross-sectional design precluded causal inference between study variables. However, it seems less likely that mental health and preventive behaviors drive exposure to social capital. Second, a self-report diagnostic screening rather than clinical interview was used to assess depressive symptoms in this study. We note however that the instruments we used for assessing probable depression were well-validated and widely used with established norms in Greater China [31,40], and it is practically impossible to conduct face-to-face interviews among representative samples of a population during a pandemic. Third, we assessed each of the perceived aspects of social capital (interpersonal trust, social harmony, and sense of belonging) with a single item, although these single-item scales reduced assessment load and were easy to understand. Fourth, similar to prior evidence obtained in Hong Kong [11], there was a ceiling effect for preventive behaviors including used face masks (> 97%) and washed hands more often (> 92%) in this study. Although there is increasing evidence of the effectiveness of using face masks for containing the infections, our findings on the use of face masks could be less applicable to some Western societies with different official advices and social norms/beliefs on the use of face masks [55]. Fifth, while there were gender and age differences in our analyzes, these differences were taken into account in our statistical analysis when addressing each of our study aims. Sixth, a comprehensive list of common non-pharmaceutical preventive behaviors was not assessed in our surveys due to the limitation on the protocol length of telephone survey.

## 5. Conclusions

This study offers some of the first findings on the associations of social capital with psychobehavioral responses during the current COVID-19 pandemic, stratified by changes in income since the outbreak. The present results support a recent call for governments and communities around the world to enhance social capital and thus collective efficacy [56]. Change in socioeconomic resources, especially income loss, should be taken into account when evaluating associations between social capital and common psychiatric conditions such as depression, as well as when promoting preventive behaviors.

## Figures and Tables

**Table 1:** Sociodemographic characteristics of the sample (*n* = 3011) during February 25–April 29 2020.

Variable	Overall (*n* = 3011)	
	*n*	%
**Gender**		
Male	1359	45.1
Female	1652	54.9
**Age**		
15–24	441	14.6
25–34	552	18.3
35–44	554	18.4
45–54	535	17.8
55–65	455	15.1
65 or above	474	15.7
**Marital status**		
Married	1653	54.9
Unmarried/divorced/widowed	1358	45.1
**Education level**		
Tertiary or above	1485	49.3
Secondary	1289	42.8
Primary or below	237	7.9
Employment		
Employed	1781	59.1
Dependent	1106	36.7
Unemployed	124	4.1
**Monthly household income (HK$)**		
$80,000 or above	445	14.8
$60,000–$79,999	263	8.7
$40,000–$59,999	648	21.5
$20,000–$39,999	886	29.4
$19,999 or below	769	25.5
**Income change**		
Gain/No change	2096	69.6
Loss	915	30.4

**Table 2:** Prevalence of probable depression, adoption of preventive behaviors, and social capital.

Variable	Overall (*n* = 3011)
**Social Capital ^1^**	
Interpersonal trust	2012 (66.8%, 65.1–68.5%)
Social harmony	1652 (54.9%, 53.1–56.6%)
Sense of belonging	2908 (96.6%, 95.9–97.2%)
**Probable depression ^2,3^**	642 (21.3%, 19.9–22.8%)
**Adoption of preventive behaviors ^4^**	
Used face masks	2940 (97.6%, 97–98%)
Washed hands more often	2778 (92.3%, 91–93%)
Avoided contact with people with respiratory symptoms	2171 (72.1%, 70–74%)
Avoided going to crowded places	2399 (79.7%, 78–81%)

Data are *n* (%, 95% confidence interval). ^1^ Number and proportion represent respondents reporting presence of social capital. ^2^ Number and prevalence represent respondents that had probable depression. ^3^ The 9-item Patient Health Questionnaire (PHQ-9) scores equal to or exceeding 10 were used to define probable depression. ^4^ Number and proportion represent the adoption of preventive behaviors.

**Table 3:** Multivariable logistic regression examining the associations of social capital with probable depression and adoption of preventive behaviors.

Variable	Probable Depression ^1^	Used Face Masks	Washed Hands More Often	Avoided Contact with People with Respiratory Symptoms	Avoided Going to Crowded Places
	aOR (95% CI)	aOR (95% CI)	aOR (95% CI)	aOR (95% CI)	aOR (95% CI)
**Gender**					
Male	1.0	1.0	1.0	1.0	1.0
Female	1.55 (1.28–1.87) ***	1.70 (1.03–2.81) *	2.35 (1.76–3.15) ***	1.57 (1.32–1.87) ***	1.76 (1.46–2.12) ***
**Age**					
15–24	1.0	1.0	1.0	1.0	1.0
25–34	1.20 (0.85–1.70)	0.13 (0.03–0.61) *	1.00 (0.56–1.77)	0.91 (0.64–1.31)	1.30 (0.91–1.85)
35–44	1.15 (0.79–1.67)	0.30 (0.05–1.69)	1.10 (0.60–2.01)	0.66 (0.45–0.95) *	1.63 (1.11–2.41) *
45–54	0.91 (0.62–1.33)	0.14 (0.03–0.70) *	1.19 (0.65–2.19)	0.49 (0.34–0.71) ***	1.39 (0.95–2.04)
55–64	0.87 (0.60–1.27)	0.10 (0.02–0.46) **	0.74 (0.43–1.28)	0.42 (0.30–0.60) ***	1.26 (0.86–1.84)
65 or above	1.03 (0.70–1.51)	0.08 (0.02–0.37) **	0.92 (0.52–1.62)	0.44 (0.31–0.64) ***	1.28 (0.86–1.90)
**Marital status**					
Married	1.0	1.0	1.0	1.0	1.0
Unmarried/divorced/widowed	1.37 (1.11–1.69) **	0.82 (0.48–1.40)	0.81 (0.58–1.12)	0.81 (0.67–0.99) *	0.90 (0.72–1.12)
**Education level**					
Tertiary or above	1.0	1.0	1.0	1.0	1.0
Secondary	1.28 (1.04–1.59) *	0.66 (0.36–1.21)	0.37 (0.26–0.52) ***	0.61 (0.50–0.74) ***	0.67 (0.54–0.83) ***
Primary or below	1.56 (1.06–2.31) *	0.64 (0.27–1.52)	0.28 (0.16–0.48) ***	0.40 (0.29–0.57) ***	0.40 (0.27–0.59) ***
**Employment**					
Employed	1.0	1.0	1.0	1.0	1.0
Dependent	1.09 (0.85–1.41)	0.53 (0.26–1.06)	0.88 (0.60–1.29)	1.13 (0.89–1.42)	1.61 (1.24–2.09) ***
Unemployed	1.80 (1.20–2.71) **	0.35 (0.13–0.92) *	0.94 (0.48–1.84)	1.18 (0.76–1.82)	1.21 (0.76–1.93)
**Monthly household income (HK$)**					
$80,000 or above	1.0	1.0	1.0	1.0	1.0
$60,000–$79,999	0.92 (0.58–1.44)	0.84 (0.26–2.71)	0.74 (0.36–1.49)	0.80 (0.55–1.17)	0.66 (0.44–0.99) *
$40,000–$59,999	1.66 (1.18–2.33) **	0.74 (0.29–1.86)	0.65 (0.37–1.15)	0.80 (0.59–1.09)	0.64 (0.46–0.90) *
$20,000–$39,999	1.64 (1.18–2.27) **	0.95 (0.38–2.37)	0.68 (0.39–1.18)	0.62 (0.46–0.83) **	0.58 (0.42–0.80) **
$19,999 or below	1.52 (1.07–2.16) *	0.98 (0.39–2.46)	0.63 (0.35–1.10)	0.64 (0.47–0.87) **	0.60 (0.42–0.86) **
**Income change**					
Gain/No change	1.0	1.0	1.0	1.0	1.0
Loss	1.35 (1.11–1.64) **	1.25 (0.71–2.20)	1.09 (0.80–1.48)	1.25 (1.04–1.51) *	1.26 (1.02–1.54) *
**Social capital**					
Presence of interpersonal trust	1.0	1.0	1.0	1.0	1.0
Lack of interpersonal trust	1.58 (1.29–1.93) ***	0.66 (0.38–1.15)	0.87 (0.64–1.19)	1.28 (1.06–1.56) *	0.95 (0.77–1.16)
Presence of social harmony	1.0	1.0	1.0	1.0	1.0
Lack of social harmony	1.17 (0.96–1.43)	1.40 (0.81–2.42)	1.03 (0.76–1.40)	0.92 (0.76–1.10)	0.95 (0.78–1.16)
Presence of sense of belonging	1.0	1.0	1.0	1.0	1.0
Lack of sense of belonging	2.53 (1.66–3.84) ***	0.31 (0.13–0.74) **	0.44 (0.25–0.80) **	0.47 (0.31–0.73) ***	0.58 (0.37–0.91) *

^1^ Scores of 10 or above in the 9-item Patient Health Questionnaire (PHQ-9) were used to define probable depression. aOR: adjusted odds ratio; CI: confidence interval. * *p* < 0.05, ** *p* < 0.01, *** *p* < 0.001.

## Supplementary Materials

The following are available online at https://www.mdpi.com/1660-4601/17/23/8888/s1, Table S1: Multivariable logistic regression examining the associations of social capital with probable depression and adoption of preventive behaviors among respondents aged 18 or above (n = 2921), Table S2: Interaction effects of social capital and income change on probable depression and adoption of preventive behaviors among respondents aged 18 or above (n = 2921).

## Appendix A

**Table A1:** Interaction effects of social capital and income change on probable depression and adoption of preventive behaviors.

Outcomes	Interaction Terms	*p*-Value
Probable depression		
	Interpersonal trust × Income change	0.856
	Social harmony × Income change	0.026
	Sense of belonging × Income change	0.300
Used face masks		
	Interpersonal trust × Income change	0.336
	Social harmony × Income change	0.043
	Sense of belonging × Income change	0.335
Washed hands more often		
	Interpersonal trust × Income change	0.086
	Social harmony × Income change	0.363
	Sense of belonging × Income change	0.311
Avoided contact with people with respiratory symptoms		
	Interpersonal trust × Income change	0.088
	Social harmony × Income change	0.903
	Sense of belonging × Income change	0.647
Avoided going to crowded places		
	Interpersonal trust × Income change	0.880
	Social harmony × Income change	0.368
	Sense of belonging × Income change	0.603
