# Supplementary material for: Social Capital, Income Loss, and Psychobehavioral Responses amid COVID-19: A Population-Based Analysis
Source: Int J Environ Res Public Health. 2020 Nov 29;17(23):8888. doi: 10.3390/ijerph17238888 (PMC7730633; doi:10.3390/ijerph17238888)
Supplement: Supplementary file 1 [file ijerph-17-08888-s001.pdf]

International Journal of
Environmental Research and Public Health

MDPI

Supplementary Material 1

Table S1. Multivariable logistic regression examining the associations of social capital with probable depression and adoption of preventive behaviors among respondents aged 18 or above (n = 2921).

	Probable depression [1]	Use face masks	Wash hands more often	Avoid contact with people with respiratory symptoms	Avoid going to crowded places
	aOR (95% CI)	aOR (95% CI)	aOR (95% CI)	aOR (95% CI)	aOR (95% CI)
Gender					
Male	1.0	1.0	1.0	1.0	1.0
Female	1.51 (1.24–1.83)***	1.71 (1.04–2.82)*	2.46 (1.82–3.32)***	1.59 (1.34–1.89)***	1.78 (1.47–2.15)***
Age					
18–24	1.0	1.0	1.0	1.0	1.0
25–34	1.14 (0.80–1.63)	0.17 (0.04–0.82)*	1.01 (0.55–1.84)	1.01 (0.70–1.46)	1.34 (0.93–1.93)
35–44	1.09 (0.74–1.60)	0.40 (0.07–2.29)	1.12 (0.59–2.11)	0.73 (0.50–1.07)	1.70 (1.14–2.53)*
45–54	0.85 (0.57–1.26)	0.19 (0.04–0.96)*	1.22 (0.64–2.31)	0.55 (0.38–0.81)**	1.45 (0.97–2.16)
55–64	0.80 (0.54–1.20)	0.13 (0.03–0.64)*	0.76 (0.42–1.38)	0.48 (0.33–0.70)***	1.32 (0.89–1.97)
65 or above	0.93 (0.62–1.40)	0.11 (0.02–0.51)**	0.95 (0.51–1.78)	0.51 (0.35–0.75)**	1.36 (0.89–2.07)
Marital status					
Married	1.0	1.0	1.0	1.0	1.0
Unmarried/divorced/widowed	1.39 (1.12–1.72)**	0.82 (0.48–1.40)	0.81 (0.58–1.12)	0.81 (0.67–0.99)*	0.91 (0.73–1.14)
Education level					
Tertiary or above	1.0	1.0	1.0	1.0	1.0
Secondary	1.34 (1.07–1.66)*	0.64 (0.35–1.18)	0.37 (0.26–0.53)***	0.59 (0.48–0.72)***	0.66 (0.53–0.83)***
Primary or below	1.61 (1.09–2.39)*	0.62 (0.26–1.48)	0.29 (0.16–0.50)***	0.41 (0.29–0.58)***	0.40 (0.27–0.60)***

Employment					
Employed	1.0	1.0	1.0	1.0	1.0
Dependent	1.13 (0.88–1.45)	0.52 (0.26–1.05)	0.84 (0.57–1.24)	1.10 (0.87–1.39)	1.59 (1.22–2.07)**
Unemployed	1.78 (1.18–2.68)**	0.35 (0.13–0.93)*	0.96 (0.49–1.87)	1.21 (0.78–1.88)	1.22 (0.76–1.95)
Monthly household income (HK$)					
$80,000 or above	1.0	1.0	1.0	1.0	1.0
$60,000–$79,999	0.87 (0.54–1.38)	0.84 (0.26–2.73)	0.75 (0.36–1.59)	0.79 (0.54–1.16)	0.67 (0.44–1.03)
$40,000–$59,999	1.68 (1.19–2.37)**	0.74 (0.29–1.89)	0.59 (0.32–1.06)	0.78 (0.57–1.06)	0.64 (0.45–0.90)*
$20,000–$39,999	1.65 (1.18–2.30)**	0.96 (0.39–2.40)	0.64 (0.36–1.13)	0.61 (0.45–0.81)**	0.58 (0.42–0.81)**
$19,999 or below	1.54 (1.08–2.21)*	0.99 (0.39–2.49)	0.58 (0.32–1.05)	0.60 (0.44–0.83)**	0.58 (0.40–0.83)**
Income change					
Gain/No change	1.0	1.0	1.0	1.0	1.0
Loss	1.35 (1.11–1.65)**	1.25 (0.71–2.20)	1.07 (0.79–1.47)	1.24 (1.03–1.50)*	1.30 (1.05–1.60)*
Social capital					
Presence of interpersonal trust	1.0	1.0	1.0	1.0	1.0
Lack of interpersonal trust	1.53 (1.24–1.88)***	0.67 (0.38–1.15)	0.84 (0.61–1.16)	1.28 (1.05–1.56)*	0.95 (0.77–1.17)
Presence of social harmony	1.0	1.0	1.0	1.0	1.0
Lack of social harmony	1.17 (0.96–1.44)	1.4 (0.81–2.42)	1.10 (0.81–1.51)	0.92 (0.77–1.11)	0.95 (0.77–1.16)
Presence of sense of belonging	1.0	1.0	1.0	1.0	1.0
Lack of sense of belonging	2.58 (1.68–3.96)***	0.31 (0.13–0.74)**	0.41 (0.22–0.74)**	0.44 (0.29–0.69)***	0.57 (0.36–0.91)*

[1] Scores of 10 or above in the 9-item Patient Health Questionnaire (PHQ-9) were used to define probable depression. aOR, adjusted odds ratio. CI, confidence interval. * $p < 0.05$, ** $p < 0.01$, *** $p < 0.001$

International Journal of
Environmental Research and Public Health

MDPI

Table S2. Interaction effects of social capital and income change on probable depression and adoption of preventive behaviors among respondents aged 18 or above (n = 2921).

**Outcomes**	**Interaction terms**	***p* value**
Probable depression		
	Interpersonal trust × Income change	0.949
	Social harmony × Income change	0.043
	Sense of belonging × Income change	0.314
Use face masks		
	Interpersonal trust × Income change	0.340
	Social harmony × Income change	0.041
	Sense of belonging × Income change	0.340
Wash hands more often		
	Interpersonal trust × Income change	0.161
	Social harmony × Income change	0.331
	Sense of belonging × Income change	0.461
Avoid contact with people with respiratory symptoms		
	Interpersonal trust × Income change	0.154
	Social harmony × Income change	0.991
	Sense of belonging × Income change	0.450
Avoid going to crowded places		
	Interpersonal trust × Income change	0.850
	Social harmony × Income change	0.296
	Sense of belonging × Income change	0.535
